# Do Alzheimer Disease genetic loci influence the risk of Age‐Related Macular Degeneration?

**DOI:** 10.1002/alz.089652

**Published:** 2025-01-03

**Authors:** Noel C. Moore, Yeunjoo E. Song, Audrey Lynn, Kristy L. Miskimen, Weihuan Wang, Renee A. Laux, Sarada L. Fuzzell, Sherri D. Hochstetler, Laura J. Caywood, Jason E. Clouse, Sharlene D. Herington, Muneeswar Gupta Nittala, SriniVas R Sadda, Dwight Stambolian, Alan J. Lerner, Jeffery M. Vance, Michael L. Cuccaro, William K. Scott, Margaret A. Pericak‐Vance, Jonathan L. Haines

**Affiliations:** ^1^ Department of Population and Quantitative Health Sciences, Case Western Reserve University School of Medicine, Cleveland, OH USA; ^2^ Department of Population and Quantitative Health Sciences, Case Western Reserve University, Cleveland, OH USA; ^3^ Cleveland Institute for Computational Biology, Case Western Reserve University, Cleveland, OH USA; ^4^ John P. Hussman Institute for Human Genomics, University of Miami Miller School of Medicine, Miami, FL USA; ^5^ Doheny Eye Institute, University of California, Los Angeles, CA USA; ^6^ Department of Ophthalmology, University of Pennsylvania, Philadelphia, PA USA; ^7^ Department of Neurology, University Hospitals Cleveland Medical Center, Cleveland, OH USA; ^8^ Case Western Reserve University School of Medicine, Cleveland, OH USA; ^9^ Dr. John T. Macdonald Foundation Department of Human Genetics, University of Miami Miller School of Medicine, Miami, FL USA

## Abstract

**Background:**

Alzheimer’s Disease (AD) and Age‐Related Macular Degeneration (AMD) are two age related neurodegenerative diseases that share multiple characteristics, including deposition of amyloid beta. In AD, amyloid plaque accumulation contributes to neurological dysfunction, while in AMD amyloid is a component of the hallmark retinal drusen complexes that lead to degeneration of central vision. Both diseases have significant and opposite risk due to the APOE e4 and e2 alleles. Investigation into other potential genetic similarities between the two diseases is warranted.

**Methods:**

Variants from the recent AMD meta‐analysis (Fritsche et al., 2016) and AD meta‐analysis (Bellenguez et al., 2022) were utilized to generate genetic risk scores (GRS) in an Amish founder population. 1293 individuals (1120 controls and 173 cases) were included in the AD analysis and 654 individuals were included in the AMD analysis (334 controls and 320 cases). AMD‐only and AD‐only GRSs were applied separately to both AMD and AD. We then combined both the AMD and AD variants into a single GRS applied to each disease. We subsequently removed AD SNPs sequentially from the GRS to identify any AD variants that might improve the association with AMD.

**Results:**

As expected, the AD‐only GRS successfully predicted AD (p = 0.01). However, the AMD‐only GRS was less successful in predicting AMD (p = 0.145). The combined AD/AMD GRS was more successful in predicting AMD (p = 0.12) than the AMD‐only GRS, while AD prediction decreased in performance (p = 0.67) compared to the AD‐only GRS. The sequential analysis identified thirteen AD variants that improved the AMD‐only GRS when combined with the AMD variants (p = 0.06).

**Conclusions:**

While the AD‐only GRS was most successful in predicting AD, the AD/AMD GRS outperformed the AMD‐only GRS in predicting AMD. These results suggest that several AD loci in addition to APOE may also influence AMD.

